# COVID-19 and risk of self-harm, suicidal ideation, and poisoning in children and adolescents

**DOI:** 10.1080/07853890.2025.2516698

**Published:** 2025-06-09

**Authors:** Jaewhan Kim, Colby Hoskinson, Evelyn Nichols, Chathuri Illapperuma-Wood, Aaron Fischer, Kenechukwu Ben-Umeh, Fernando Wilson

**Affiliations:** a Department of Physical Therapy, University of Utah, Salt Lake City, UT, USA; b Department of Educational Psychology, University of Utah, Salt Lake City, UT, USA; c Department of Pharmacotherapy, University of Utah, Salt Lake City, UT, USA; d Department of Population Health Sciences, University of Utah, Salt Lake City, UT, USA

**Keywords:** Self-harm, Suicidal ideation, Poisoning, Children, Adolescents, COVID-19

## Abstract

**Objective:**

This study examined the association between COVID-19 and the risk of self-harm, suicidal ideation and poisoning in school-aged children and adolescents.

**Materials and methods:**

We utilized the 2019–2021 Utah All Payers Claims Database (APCD) to identify school-age children and adolescents aged 6–15 years in 2019. COVID-19 diagnosis, self-harm, suicidal ideation and poisoning were identified using ICD-10 diagnosis codes. Baseline characteristics were assessed, and Inverse Probability Weighting (IPW) was applied to balance these characteristics between the two groups (those with COVID-19 and those without). Weighted logistic regression was employed to identify factors associated with these outcomes in 2021.

**Results:**

The study included 180,925 participants (48% female; mean [SD] age in 2019: 10.6 [2.9] years), of whom 45,056 (24.9%) had a COVID-19 in 2020, and 51.5% were aged 11–15 years. Subjects with COVID-19 had twice the odds of suicidal ideation (AOR = 2.00, *p* < 0.01) and more than twice the odds of self-harm (AOR = 2.05, *p* < 0.01) and poisoning (AOR = 2.21, *p* < 0.01) compared to those without COVID-19. Adolescents aged 11–15 years had nearly four times the odds of suicidal ideation compared to those aged 6–10 years (AOR = 3.97, *p* < 0.01) while female participants were significantly more likely to engage in self-harm than male participants (AOR = 1.73, *p* < 0.01).

**Conclusion:**

Our findings suggest a significant association between diagnosed COVID-19 and self-harming behaviours in children and adolescents.

## Introduction

The COVID-19 pandemic introduced a novel public health concern across the globe. From controlling infection rates to addressing psychological and socioeconomic concerns stemming from lockdowns and social isolation, the world faced challenges across a wide variety of domains [1,2]. Among children and adults, over 57 million cases were recorded worldwide with prevalence rates varying from 0% to 37% across countries [3]. While infections in this demographic were predominantly asymptomatic or mild [3,4], children and adolescents were disproportionately affected by mental health outcomes, including self-harm, ­suicidal ideation and other related behaviours [5,6].

Self-harm, a leading cause of death among ­adolescents and youths, encompasses a spectrum of behaviours, such as cutting, deliberate self-poisoning and attempted suicide [7,8]. Globally, the pooled ­prevalence of self-harm among adolescents is approximately 18% [9], with risk factors including socioeconomic disadvantage, behavioural disorders, personality disorders, depression and anxiety [8,10]. Evidence suggests that the COVID-19 pandemic exacerbated these risks due to disruptions to schooling, social life and extracurricular activities [5]. For instance, a cohort study using emergency department data from 25 countries found that the rate of self-harm doubled between 2020 and 2021, and was 1.7 times higher compared to pre-pandemic levels in 2019 [11].

Beyond its well-documented somatic symptoms—fever, cough, shortness of breath and loss of taste or smell—the psychological impact of COVID-19, particularly in children and adolescents, remains unclear [12]. Preliminary findings indicate that COVID-19, like other infections, can cause neuroinflammation, potentially leading to symptoms such as brain fog, sleep disturbances, anxiety, stress, depression and suicidal ideation [13–16]. At the brink of the pandemic in 2019, suicide was already the second leading cause of death in children between ages 10 and 17 years old in the United States [17,18]. During the pandemic, emergency departments also reported significantly higher rates of unintentional, intentional (self-harm) and recreational drug poisoning in children and teenagers between ages 0 to 18 years old, with the latter two being the primary reasons for teen poisoning.

While much of the extant literature highlights behavioural health outcomes in children and adolescents related to the large-scale lockdowns and social isolation, limited studies have explored the direct impact of COVID-19 on self-harm, suicidal ideation and poisoning in this population. Research in adults has highlighted a potential association between COVID-19 and suicidal behaviour [12,19,20]. A systematic review by Sinyor and colleagues reported an association between COVID-19 and suicidal ideation and self-harm in adults, with a higher prevalence in individuals who were infected compared to those who were not [21]. Similarly, studies also reported the incidence of chemical poisoning such as methanol ingestion, stemming from misinformation about its protective effects against COVID-19 [22].

Despite these findings, there is a notable research gap on the impacts of COVID-19 on self-harm, suicidal ideation and poisoning collectively in children and adolescents. To address this gap, this study uses population-level data to examine the relationship between COVID-19 and the risk of self-harm, suicidal ideation and poisoning in school-aged children and adolescents during the pandemic.

## Materials and methods

### Data

This study used the 2019–2021 Utah All Payers Claims Database (APCD), which covers approximately 70% of the Utah population, including children, adolescents and adults. The APCD includes claims data submitted by private payers, Medicaid and Medicare Advantage. It consists of two main files: the enrolment file and the claims file. The enrollment file provides information on insurance coverage start and end dates, type of insurance (e.g. private insurance, Medicaid, or Medicare Advantage), race/ethnicity and date of birth. The claims file includes details such as visit dates, claim types (e.g. medical or pharmacy claims), International Classification of Diseases-10^th^ Revision (ICD-10) diagnosis and procedure codes, Current Procedural Terminology (CPT) codes, national drug codes (NDC), reimbursed amounts, place of service and revenue codes. This database has been used extensively to examine healthcare utilization, healthcare spending, disease incidence and the impact of the pandemic. More information on the APCD is available from prior research [23,24]. This study was approved by the University of Utah Institutional Review Board (IRB 00151091) with exemption, as it used de-identified data. Patient consent was waived for this study because it poses minimal risk to participants and aligns with ethical guidelines for research.

### Study population

School-age children and adolescents aged 6–15 years in 2019 were included in the study. Adolescents who were 15 years old in 2019 and had reached 17 years old by 2021, but were still in high school, were also selected. Individuals without continuous insurance coverage from 2019 to 2021 were excluded. Additionally, individuals who had a recorded COVID-19 in 2021 were excluded from the study.

### Outcomes

Suicidal ideation, self-harm and poisoning were the outcomes of this study, identified using ICD-10 diagnosis codes based on the Agency for Healthcare Research and Quality (AHRQ) Clinical Classifications Software Refined (CCSR) [25]. The CCSR maps over 70,000 ICD-10 diagnosis codes into approximately 530 clinically meaningful categories for 22 body systems [25]. Suicidal ideation was identified using code R45.851, categorized under the Suicidal ideation/Attempt category in the CCSR, which reflects documented ideation in clinical records. Self-harm was identified using external cause codes ranging from X71 through X83, categorized under External Cause Codes in the CCSR. Poisoning was identified using ICD-10 codes T36 through T65, drawn from the Poisoning by Drugs and Poisoning/Toxic Effect categories in the CCSR. Each outcome variable was binary (Yes/No). In addition, an overall harm variable combining the three outcomes (i.e. subjects with at least one of the outcomes) was created to capture subjects who experienced any of the three outcomes. This combined measure was coded as binary (Yes/No) and was used to evaluate overall rates of harm during the pandemic.

### Independent variable

Having COVID-19 (Yes/No) in 2020 was the independent variable. COVID-19 was identified using ICD-10 diagnosis codes (e.g. J12.81, U07.1) and CPT codes (86413, 86328) [25,26]. Among participants with a confirmed COVID-19, those who were admitted to the hospital or visited the emergency room (ER) within 7 days before or after their COVID-19 diagnosis were categorized as having severe COVID-19. Conversely, participants with COVID-19 who had no hospital admission or ER visits were categorized as having moderate COVID-19 [23].

### Covariates

Variables potentially associated with self-harm, suicidal ideation and poisoning were included as covariates in the regression model [8–10]. These variables include age in 2019 (6–10 vs. 11–15 years old), sex (male/female), race/ethnicity (non-Hispanic White, Hispanic, non-Hispanic Black, non-Hispanic Asian/American Indian/Pacific Islander and unknown) and insurance type (private insurance vs. Medicaid). The analysis also accounted for comorbid conditions, including sleep disorders, attention-deficit/hyperactivity disorder (ADHD), anxiety, depression, chronic pain, any injury, any fracture, obesity, any cancer, asthma, headache/migraine, severe allergies and neurologic disorders, all coded as binary variables (Yes/No). All ICD-10 diagnosis codes for the comorbid conditions were sourced from the AHRQ CCSR [25]. Additionally, baseline measures of suicidal ideation, self-harm and poisoning diagnosed in 2019 were included to control for their potential influence on the incidence of these outcomes in 2021.

### Statistical approach

Because baseline characteristics (i.e. 2019) of participants with and without COVID-19 were statistically different, Inverse Probability Weighting (IPW) was applied to balance these characteristics between the two groups. IPW was calculated using logistic regression, where the outcome was COVID-19 (Yes or No). All participants who met the inclusion criteria were included in the IPW calculation. All covariates described in the Covariates section were used in the IPW estimation, and these variables are listed in Appendix A. Standardized mean differences (SMD) were used to assess statistical differences in the baseline characteristics between the two groups. SMD less than 0.01 indicates no meaningful differences between the groups [27]. Before applying IPW, all covariates had SMDs greater than 0.01, indicating significant baseline differences. After IPW, all covariates had SMDs close to 0 (<0.01), indicating effective balancing (Appendix A). Weighted summary statistics, including means, standard deviations and percentages, were used to describe the characteristics of the study population. As the outcomes were binary, weighted multiple logistic regression was employed to identify factors associated with the outcomes. A *p*-value less than 0.05 was considered statistically significant. Analyses were performed using Stata, version18.5 (StataCorp, College Station, TX).

## Results

In 2019, a total of 352,087 children aged 0 to 17 years with continuous enrollment from 2019 to 2021 were identified. Of these, 217,434 children aged 6 to 15 in 2019 were selected, and 36,509 participants with COVID-19 in 2021 were excluded. The final study population included 180,925 participants. Within this population, 45,056 (24.90%) had COVID-19 in 2020. The average age in 2019 was 10.6 years (2.9), with 51.5% in the 11–15 age group. Additionally, 48% were female, and 32% were covered by Medicaid. Among the participants, 2.7% had a sleep disorder, 6.8% had ADHD and 9.4% had an anxiety diagnosis (Table 1).

**Table 1.** Baseline characteristics of children and adolescents in 2019, with and without COVID-19.

	COVID-19			
Variable	No	Yes	Total	Standardized mean difference (SMD)
	135,869 (75.1%)	45,056 (24.9%)	180,925 (100.0%)	
	Mean (sd)/%	Mean (sd)/%	Mean (sd)/%	
Age in 2019 as continuous	10.552 (2.818)	10.641 (2.880)	10.597 (2.849)	−0.031
Age category				−0.002
6–10 years old	65,761 (48.40%)	21,852 (48.50%)	87,749 (48.50%)	
11–15 years old	70,108 (51.60%)	23,204 (51.50%)	93,176 (51.50%)	
Female	65,489 (48.20%)	21,627 (48.00%)	87,025 (48.10%)	−0.004
Race/Ethnicity				0.001
Non-Hispanic white	21,196 (15.60%)	6,984 (15.50%)	28,043 (15.50%)	
Hispanic	12,500 (9.20%)	4,145 (9.20%)	16,645 (9.20%)	
Non-Hispanic black	1,359 (1.00%)	451 (1.00%)	1,809 (1.00%)	
Non-Hispanic others*	2,310 (1.70%)	811 (1.80%)	3,257 (1.80%)	
Unknown	98,505 (72.50%)	32,666 (72.50%)	131,171 (72.50%)	
Medicaid	43,886 (32.30%)	14,508 (32.20%)	58,439 (32.30%)	−0.003
Baseline comorbid conditions				
Sleep disorder	3,668 (2.70%)	1,262 (2.80%)	4,885 (2.70%)	0.003
ADHD	8,967 (6.60%)	3,064 (6.80%)	12,122 (6.70%)	0.006
Anxiety disorder	12,636 (9.30%)	4,235 (9.40%)	16,826 (9.30%)	0.004
Depression	6,250 (4.60%)	2,118 (4.70%)	8,503 (4.70%)	0.002
Chronic pain	20,788 (15.30%)	6,939 (15.40%)	27,862 (15.40%)	0.002
Injury	12,772 (9.40%)	4,280 (9.50%)	17,007 (9.40%)	0.003
Suicidal ideation	815 (0.60%)	270 (0.60%)	1,086 (0.60%)	−0.002
Self-harm	1,359 (1.00%)	451 (1.00%)	1,809 (1.00%)	0.005
Poisoning	272 (0.20%)	90 (0.20%)	362 (0.20%)	0.006
Obesity	2,853 (2.10%)	946 (2.10%)	3,799 (2.10%)	0.002
Cancer	7,609 (5.60%)	2,523 (5.60%)	10,132 (5.60%)	0.002
Asthma	6,929 (5.10%)	2,298 (5.10%)	9,227 (5.10%)	0.003
Chronic headache/migraine	4,620 (3.40%)	1,577 (3.50%)	6,332 (3.50%)	0.003
Any allergy	10,326 (7.60%)	3,469 (7.70%)	13,931 (7.70%)	0.004
Neurologic disorders	12,772 (9.40%)	4,325 (9.60%)	17,188 (9.50%)	0.006

*Non-Hispanic others included non-Hispanic Asian/American Indian/Pacific Islander.

In 2021, suicidal ideation was reported by 2.1% of participants with COVID-19, compared to 0.7% among those without COVID-19 (*p* < 0.01). Similarly, self-harm was observed in 2.3% of participants with COVID-19 versus 0.8% for those without (*p* < 0.01). The proportion of participants who experienced poisoning was also higher among those with COVID-19 (0.6%) compared to those without (0.2%, *p* < 0.01).

Participants with severe COVID-19 had higher rates of suicidal ideation (3.1% vs. 0.7%; *p* < 0.01), self-harm (3.3% vs. 0.8%; *p* < 0.01) and poisoning (0.9% vs. 0.2%; *p* < 0.01) compared to those without COVID-19. Furthermore, when compared to participants with moderate COVID-19, those with severe COVID-19 had higher rates of suicidal ideation (3.1% vs. 1.1%; *p* < 0.01), self-harm (3.3% vs. 1.3%; *p* < 0.01) and poisoning (0.9% vs. 0.4%; *p* < 0.01) (Table 2).

**Table 2.** Incidence of self-harm, suicidal ideation, and poisoning in 2021, with and without COVID-19.

	COVID-19				
	No	Yes	Total	*p*-value	
N	135,869 (75.1%)	45,056 (24.9%)	180,925 (100.0%)		
Suicidal ideation	991 (0.7%)	925 (2.1%)	1,916 (1.1%)	<0.001	
Self-harm	1,127 (0.8%)	1,015 (2.3%)	2,142 (1.2%)	<0.001	
Poisoning	286 (0.2%)	286 (0.6%)	572 (0.3%)	<0.001	
Overall self-harm	1,778 (1.3%)	1,498 (3.3%)	3,276 (1.8%)	<0.001	
	Severity of COVID-19				
	No infection	Moderate infection	Severe infection	Total	*p*-value
N	135,869 (75.1%)	23,023 (12.7%)	22,033 (12.2%)	180,925 (100.0%)	
Suicidal ideation	991 (0.7%)	251 (1.1%)	674 (3.1%)	1,916 (1.1%)	<0.001
Self-harm	1,127 (0.8%)	293 (1.3%)	722 (3.3%)	2,142 (1.2%)	<0.001
Poisoning	286 (0.2%)	84 (0.4%)	202 (0.9%)	572 (0.3%)	<0.001
Overall self-harm	1,778 (1.3%)	443 (1.9%)	1,055 (4.8%)	3,276 (1.8%)	<0.001

Participants with COVID-19 were twice as likely to have suicidal ideation compared to those without COVID-19 (adjusted odds ratio (AOR) = 2.00, *p* < 0.01). Adolescents aged 11–15 had nearly four times the odds of suicidal ideation compared to those aged 6–10 (AOR = 3.97, *p* < 0.01). Additionally, participants covered by Medicaid had a 31% higher likelihood of suicidal ideation compared to those with private insurance (AOR = 1.31, *p* < 0.01). The odds of self-harm were more than double among participants with COVID-19 compared to those without (AOR = 2.05, *p* < 0.01). Female participants were 73% more likely to engage in self-harm than male participants (AOR = 1.73, *p* < 0.01). Furthermore, participants with COVID-19 had 2.21 times the odds of poisoning compared to those without COVID-19 (AOR = 2.21, *p* < 0.01), and female participants had 2.03 times higher odds of poisoning than males (AOR = 2.03, *p* < 0.01) (Table 3).

**Table 3.** Factors associated with self-harm, suicidal ideation, and poisoning in children and adolescents (*n* = 180,925).

	Suicidal ideation				Self-harm			
Covariate	Odds ratio	*p*-value	95% CI		Odds ratio	*p*-value	95% CI	
COVID-19	2.00	<0.01	1.81	2.20	2.05	<0.01	1.87	2.24
Age category								
6–10 years old	Reference				Reference			
11–15 years old	3.97	<0.01	3.41	4.64	2.26	<0.01	2.00	2.56
Female	2.06	<0.01	1.84	2.30	1.73	<0.01	1.56	1.92
Race/Ethnicity								
Non-Hispanic white	Reference				Reference			
Hispanic	0.69	<0.01	0.57	0.83	0.81	0.03	0.67	0.97
Non-Hispanic black	0.72	0.16	0.45	1.15	0.80	0.45	0.45	1.42
Non-Hispanic others	0.89	0.53	0.61	1.29	0.84	0.33	0.59	1.19
Unknown	0.59	<0.01	0.51	0.69	0.68	<0.01	0.59	0.79
Medicaid	1.31	<0.01	1.13	1.52	1.28	<0.01	1.11	1.47
Baseline comorbid conditions								
Sleep disorder	1.11	0.28	0.92	1.35	1.18	0.09	0.97	1.44
ADHD	1.62	<0.01	1.19	2.20	1.19	0.22	0.90	1.56
Anxiety disorder	2.45	<0.01	2.11	2.84	1.56	<0.01	1.34	1.80
Depression	2.18	<0.01	1.85	2.56	1.93	<0.01	1.64	2.27
Chronic pain	1.12	0.07	0.99	1.27	1.26	<0.01	1.12	1.42
Injury	1.22	0.01	1.06	1.41	1.30	<0.01	1.13	1.49
Suicidal ideation	2.37	<0.01	1.84	3.03	0.93	0.62	0.69	1.25
Self-harm	1.36	0.04	1.01	1.84	4.28	<0.01	3.29	5.56
Poisoning	1.01	0.96	0.65	1.58	1.90	<0.01	1.32	2.74
Obesity	0.90	0.48	0.68	1.20	0.85	0.27	0.64	1.13
Cancer	0.98	0.79	0.82	1.16	1.32	<0.01	1.12	1.54
Asthma	0.98	0.80	0.81	1.17	1.04	0.66	0.87	1.24
Chronic headache/migraine	1.22	0.03	1.02	1.47	1.18	0.08	0.98	1.42
Any allergy	1.07	0.43	0.90	1.27	1.29	<0.01	1.11	1.51
Neurologic disorders	1.09	0.58	0.80	1.48	1.30	0.06	0.99	1.70
	Poisoning				Overall self-harm			
Covariate	Odds ratio	*p*-value	95% CI		Odds ratio	*p*-value	95% CI	
COVID-19	2.21	<0.01	1.85	2.63	1.91	<0.01	1.77	2.05
Age category								
6–10 years old	Reference				Reference			
11–15 years old	3.34	<0.01	2.57	4.35	2.54	<0.01	2.29	2.82
Female	2.03	<0.01	1.65	2.50	1.71	<0.01	1.57	1.86
Race/Ethnicity								
Non-Hispanic white	Reference				Reference			
Hispanic	0.91	0.57	0.66	1.26	0.76	<0.01	0.65	0.88
Non-Hispanic black	1.23	0.62	0.54	2.84	0.83	0.40	0.54	1.28
Non-Hispanic others	1.13	0.69	0.62	2.09	0.81	0.17	0.61	1.09
Unknown	0.71	0.02	0.53	0.94	0.67	<0.01	0.59	0.75
Medicaid	1.73	<0.01	1.30	2.29	1.29	<0.01	1.15	1.45
Baseline comorbid conditions								
Sleep disorder	1.07	0.69	0.76	1.51	1.15	0.09	0.98	1.35
ADHD	2.42	<0.01	1.38	4.27	1.39	0.01	1.10	1.75
Anxiety disorder	1.32	0.04	1.01	1.73	1.84	<0.01	1.63	2.07
Depression	1.81	<0.01	1.36	2.41	2.04	<0.01	1.79	2.32
Chronic pain	1.17	0.17	0.93	1.46	1.24	<0.01	1.12	1.36
Injury	1.55	<0.01	1.20	2.00	1.29	<0.01	1.15	1.44
Suicidal ideation	0.67	0.15	0.39	1.15	1.31	0.03	1.02	1.66
Self-harm	3.46	<0.01	2.06	5.81	2.94	<0.01	2.31	3.75
Poisoning	5.84	<0.01	3.38	10.08	1.63	0.01	1.14	2.34
Obesity	0.59	0.09	0.32	1.08	0.91	0.38	0.72	1.13
Cancer	1.03	0.87	0.74	1.43	1.21	<0.01	1.06	1.39
Asthma	0.98	0.92	0.71	1.36	1.01	0.92	0.87	1.17
Chronic headache/migraine	0.87	0.47	0.59	1.27	1.27	<0.01	1.10	1.48
Any allergy	1.37	0.03	1.03	1.80	1.25	<0.01	1.10	1.42
Neurologic disorders	0.93	0.80	0.53	1.63	1.27	0.04	1.01	1.59

After adjusting for confounders in the weighted logistic regressions, participants with moderate COVID-19 had 33% higher odds of experiencing suicidal ideation compared to those without COVID-19 (AOR = 1.33, *p* < 0.01), while those with severe COVID-19 had 153% higher odds (AOR = 2.53, *p* < 0.01). Similarly, participants with moderate and severe COVID-19 had 43% (AOR = 1.43, *p* < 0.01) and 157% (AOR = 2.57, *p* < 0.01) higher odds of engaging in self-harm, respectively, compared to those without COVID-19. For poisoning, moderate and severe COVID-19 were associated with 60% (AOR = 1.60, *p* < 0.01) and 168% (AOR = 2.68, *p* < 0.01) higher odds, respectively, compared to no COVID-19 (Table 4).

**Table 4.** Association of COVID-19 severity with self-harm, suicidal ideation, and poisoning (*n* = 180,925).

	Suicidal ideation				Self-harm			
Covariate	Odds ratio	*p*-value	95% CI		Odds ratio	*p*-value	95% CI	
Severity of COVID-19								
No infection	Reference				Reference			
Mild infection	1.33	<0.01	1.15	1.54	1.43	<0.01	1.25	1.64
Severe infection	2.53	<0.01	2.27	2.83	2.57	<0.01	2.32	2.85
Age category								
6–10 years old	Reference				Reference			
11–15 years old	3.95	<0.01	3.39	4.61	2.25	<0.01	1.99	2.54
Female	2.04	<0.01	1.83	2.28	1.72	<0.01	1.55	1.91
Race/Ethnicity								
Non-Hispanic white	Reference				Reference			
Hispanic	0.69	<0.01	0.57	0.83	0.81	0.02	0.67	0.97
Non-Hispanic black	0.70	0.14	0.44	1.12	0.78	0.41	0.44	1.39
Non-Hispanic others	0.90	0.58	0.62	1.31	0.85	0.36	0.59	1.21
Unknown	0.63	<0.01	0.54	0.73	0.72	<0.01	0.62	0.83
Medicaid	1.31	<0.01	1.13	1.51	1.28	<0.01	1.11	1.46
Baseline comorbid conditions								
Sleep	1.10	0.33	0.91	1.34	1.17	0.11	0.96	1.42
ADHD	1.65	<0.01	1.22	2.25	1.21	0.16	0.92	1.59
Anxiety disorder	2.42	<0.01	2.08	2.81	1.55	<0.01	1.33	1.79
Depression	2.13	<0.01	1.81	2.50	1.88	<0.01	1.60	2.22
Chronic pain	1.09	0.18	0.96	1.23	1.23	<0.01	1.09	1.38
Injury	1.20	0.01	1.04	1.39	1.27	<0.01	1.11	1.46
Suicidal ideation	2.33	<0.01	1.82	2.99	0.92	0.58	0.68	1.24
Self-harm	1.34	0.06	0.99	1.81	4.18	<0.01	3.22	5.43
Poisoning	1.01	0.98	0.64	1.57	1.89	<0.01	1.31	2.73
Obesity	0.90	0.45	0.68	1.19	0.84	0.23	0.63	1.12
Cancer	0.97	0.72	0.82	1.15	1.30	<0.01	1.11	1.53
Asthma	0.96	0.63	0.79	1.15	1.02	0.81	0.86	1.22
Chronic headache/migraine	1.20	0.05	1.00	1.45	1.16	0.11	0.97	1.40
Any allergy	1.07	0.47	0.90	1.27	1.28	<0.01	1.10	1.50
Neurologic disorders	1.07	0.67	0.79	1.45	1.27	0.08	0.98	1.66
	Poisoning				Overall self-harm			
Covariate	Odds ratio	*p*-value	95% CI		Odds ratio	*p*-value	95% CI	
Severity of COVID-19								
No infection	Reference				Reference			
Mild infection	1.60	<0.01	1.24	2.06	1.34	<0.01	1.20	1.50
Severe infection	2.68	<0.01	2.19	3.28	2.39	<0.01	2.19	2.60
Age category								
6–10 years old	Reference				Reference			
11–15 years old	3.33	<0.01	2.56	4.33	2.53	<0.01	2.28	2.81
Female	2.01	<0.01	1.63	2.47	1.69	<0.01	1.56	1.85
Race/Ethnicity								
Non-Hispanic white	Reference				Reference			
Hispanic	0.91	0.55	0.66	1.25	0.76	<0.01	0.65	0.88
Non-Hispanic black	1.23	0.63	0.54	2.80	0.81	0.35	0.53	1.25
Non-Hispanic others	1.15	0.65	0.63	2.12	0.82	0.19	0.61	1.10
Unknown	0.74	0.04	0.55	0.98	0.70	<0.01	0.63	0.79
Medicaid	1.72	<0.01	1.30	2.27	1.29	<0.01	1.15	1.44
Baseline comorbid conditions								
Sleep disorder	1.07	0.70	0.76	1.51	1.14	0.12	0.97	1.33
ADHD	2.46	<0.01	1.40	4.34	1.42	<0.01	1.13	1.79
Anxiety disorder	1.31	0.05	1.00	1.72	1.82	<0.01	1.62	2.05
Depression	1.77	<0.01	1.33	2.36	1.99	<0.01	1.75	2.27
Chronic pain	1.14	0.25	0.91	1.42	1.20	<0.01	1.09	1.33
Injury	1.53	<0.01	1.19	1.98	1.27	<0.01	1.13	1.42
Suicidal ideation	0.67	0.14	0.39	1.14	1.29	0.04	1.01	1.64
Self-harm	3.36	<0.01	2.00	5.65	2.88	<0.01	2.26	3.68
Poisoning	5.85	<0.01	3.39	10.11	1.62	0.01	1.13	2.33
Obesity	0.58	0.08	0.31	1.07	0.89	0.33	0.71	1.12
Cancer	1.01	0.93	0.73	1.41	1.20	0.01	1.05	1.37
Asthma	0.96	0.82	0.70	1.33	0.99	0.90	0.86	1.15
Chronic headache/migraine	0.85	0.42	0.58	1.25	1.25	<0.01	1.08	1.46
Any allergy	1.36	0.03	1.03	1.80	1.24	<0.01	1.10	1.41
Neurologic disorders	0.92	0.76	0.52	1.61	1.24	0.06	0.99	1.56

## Discussion

This population-level study utilizing the Utah All Payer Claims Database (APCD) found a significant association between COVID-19 and adverse psychological and behavioural outcomes in children and adolescents. In 2021, participants with COVID-19 demonstrated twice the odds of self-harm, suicidal ideation and poisoning compared to their counterparts without COVID-19. The observed differences were further amplified by disease severity, as those with severe COVID-19 showed higher rates and odds of self-harm, suicidal ideation and poisoning compared to those with moderate infection. These findings further highlight the potential mental health challenges associated with COVID-19 in children and adolescents.

In 2020, Utah’s COVID-19 case counts spiked beginning in early June, with 27,356 confirmed cases already reported as of 9 July 2020 [28]. By the end of the year, the state had recorded approximately 280,000 confirmed cases, translating to an average of about 970 new cases per day [29]. Despite the rise in infections, Utah maintained a lower per-capita COVID-19 mortality rate compared to the national average. From April 2020 to March 2021, Utah reported 65 deaths per 100,000 people, significantly lower than the US average of 167 deaths per 100,000. This lower mortality rate has been attributed, in part, to Utah’s relatively younger population [30]. These statistics underscore the substantial impact of COVID-19 in Utah during the study period, which may have influenced the psychological and behavioural outcomes observed in our study.

Previous studies have investigated the prevalence of psychiatric conditions during the pandemic, providing insights into trends and potential contributing factors. Wong et al. reported a twofold increase in emergency department (ED) presentations for self-harm among children and adolescents between 2020 and 2021, with a greater likelihood of such presentations occurring in girls rather than boys [11]. They partially attributed the reduced psychiatric ED presentations in 2020 to the initial restrictions from lockdowns and stay-at-home orders in various countries [11]. However, their retrospective cohort study, which spanned 25 countries, focused solely on cases resulting in ED visits and lacked comprehensive data for the entire pandemic period. In contrast, our study leveraged comprehensive population-level claims dataset covering the periods of 2019 to 2021. This dataset captured individuals covered under private insurance, Medicaid and Medicare Advantage, offering a broader perspective. Our analysis assessed the relationship between COVID-19 and the risk of self-harm, suicidal ideation and poisoning in school-aged children and adolescents. Unlike Wong et al.’s study, which primarily documented emergency cases, our study examined all reported cases, providing a comprehensive understanding of these outcomes. We also present regression analyses quantifying the odds of these outcomes between the two groups, further emphasizing the mental health burden associated with COVID-19.

Evidence on the relationship between COVID-19 and suicidal ideation remains mixed. A meta-analysis by Farooq et al. found that the pooled risk for suicidal ideation during the COVID-19 pandemic was 12.1% (CI 9.3–15.2). This rate was higher than those reported in pre-pandemic studies, suggesting a potential association with pandemic-related factors, including COVID-19 itself [31]. Similarly, a longitudinal study in the UK indicated an elevated prevalence of abuse, suicidal ideation and self-harm thoughts among individuals diagnosed with COVID-19, hinting at increased psychological risk during infection [32]. Conversely, Tsai and colleagues reported that COVID-19 alone was not independently associated with suicidal ideation. Instead, an interplay of COVID-19 positivity and comorbidities such depression, anxiety, or COVID-19 related stress, contributed to the increased association with suicidal ideation [33]. Our findings align with and expand these insights. After controlling for possible confounders such as sleep disorders, ADHD, anxiety and depression, we further observed a significant association between COVID-19 and higher odds of suicidal ideation. This was also observed for self-harm and poisoning.

The severity of COVID-19 has been highlighted to play a crucial role in the prevalence of mental health presentations [34]. Our study reinforces this as we observed that individuals with severe COVID-19 had about three times higher odds of self-harm, suicidal ideation and poisoning compared to those without COVID-19. The risk was greater when comparing severe cases to moderate cases, stressing the impact of infection severity on the observed outcome. The mechanisms underlying this association are multifaceted. COVID-19 viral strains have been shown to directly impact brain functioning and indirectly impair the body’s immune responses, potentially resulting in neuropsychiatric symptoms such as anxiety, sleep challenges, depression and suicidal ideation [35,36]. In patients with severe disease, there may also be neurocognitive impairment [35]. Depression, a critical risk factor for self-harm and suicidal ideation, has been linked to COVID-19 through immune-mediated nervous system injuries caused by infection-induced inflammatory responses [37]. It is important to note that depression has been shown to be more prevalent in adolescents [11,12]. Our study highlights this increased vulnerability, particularly for adolescents aged 11–15, who were found to be four times more likely to exhibit suicidal ideation than those aged 6–10. This indicates a demographic in need of significant mental health support and screening. Furthermore, our findings that adolescent females were more likely to engage in self-harm align with the literature, such as reported by Gracia et al. [38] By controlling for potential confounders, our study provides robust evidence on this association between COVID-19 and the outcomes explored.

To tackle these challenges, mental health screening and assessment is essential, especially among adolescents. Primary care services should encompass a thorough evaluation of the lifestyle of patients to ensure any potentially dangerous behaviour is captured early [39]. An environment that encourages open communication should also be created by the primary care provider [40]. The ‘HEADSS’ method has been recommended as a way of evaluating the wellbeing of adolescents. This refers to asking questions about the Home environment, Education and school, Eating habits, Activities (like sports and hobbies), Drugs and alcohol, Sexuality, Safety practices and mental health, including potential Suicidal thoughts or depression [41]. There is also need to expand well-structured mental health programs for children and adolescents, including insurance reimbursable telehealth services to ensure easy access [42].

Our findings are subject to several limitations. While we adjusted for different mental health conditions that could confound the relationship between COVID-19 and the outcomes studied, we may not have captured the full spectrum of these mental health conditions. More mental health comorbid conditions could be incorporated in future analyses to confirm the robustness of the observed relationship. Additionally, household-level covariates such as socio-economic status, job loss, domestic violence, social isolation, disruptions to daily routines and other stressors, were not accounted for in our data. Accounting for these factors could have provided a more robust analysis. Furthermore, our data on COVID-19 is limited by the prevalence of untested and asymptomatic cases. While the true number of COVID-19 cases in our study remains unknown, it is likely that some individuals in the control group contracted COVID-19 without being identified, particularly those who were asymptomatic. These undetected cases would predominantly be mild, as severe cases are more likely to be reported due to pronounced symptoms and the need for medical care. However, since our findings indicate more significant effects on self-harming behaviours in individuals with severe COVID-19, this limitation is unlikely to substantially affect our conclusions. COVID-19 vaccination was not widely available to children and adolescents during the study period. Although vaccines became available for individuals aged 16 and older in December 2020 and for adolescents aged 12 to 15 in May 2021, children under 12 were not eligible until late 2021 [43]. As a result, most paediatric patients in our cohort were likely unvaccinated, limiting our ability to assess the impact of vaccination on outcomes. This should be considered when interpreting the findings, particularly in the context of more recent periods when vaccination coverage among paediatric populations has increased. While cancer patients constitute 5.6% of the paediatric cohort in our study, which is higher than the typical representation of cancer patients in general paediatric populations, the sample may still be subject to selection bias. This could affect the generalizability of our results to the broader paediatric cancer population. Moreover, due to the nature of claims data, we lacked access to information on the educational experiences of participants, such as school attendance or modality (in-person vs. remote learning), academic performance, or other school-related stressors. These factors are important in determining the general wellbeing of children and adolescents. Including such variables in future analyses could strengthen the findings. Another limitation is the potential underreporting of suicidal ideation and related outcomes in administrative data. Although we used the ICD-10-CM code R45.851 to identify suicidal ideation, prior research has shown that this code may miss a substantial proportion of actual cases. Xu et al. reported that ICD-10 codes identified only about half of paediatric encounters involving suicidality, with a sensitivity of 53.4% (95% CI: 49.9%–56.9%) [44]. In our sample, only 1.1% of children and adolescents were identified as having suicidal ideation in 2021, compared to 22.3% of youth aged 14–17 in Utah’s Youth Risk Behaviour Survey reporting serious suicidal thoughts [45]. This discrepancy highlights the likelihood of under-ascertainment in claims-based data due to factors such as non-disclosure by patients, inconsistent documentation by providers, or the prioritization of other diagnoses during encounters. As a result, our findings may underestimate the true prevalence of suicidal ideation and related harms among youths during the study period. Finally, there may be limitations in the generalizability of these findings, as this study used data exclusively from the state of Utah.

## Conclusions

Our findings demonstrate a significant association between diagnosed COVID-19 and self-harming behaviours in children and adolescents. This association is further amplified by disease severity. These results are consistent with existing immunological research that links COVID-19-induced inflammation to negative psychological and mental health outcomes. These findings emphasize the critical need for robust and integrated mental health support for children and adolescents affected by COVID-19.

## Supplementary Material

Appendix A .docx

## Data Availability

Data cannot be shared publicly because of the regulations of Utah Department of Health and Human Services, and the Utah Resource for Genetic and Epidemiologic Research (RGE). Data are available from the University of Utah Institutional Data Access/Ethics Committee (contact *via* RGE) for researchers who meet the criteria for access to confidential data.
