# Supplementary material for: COVID-19 and risk of self-harm, suicidal ideation, and poisoning in children and adolescents
Source: Ann Med. 2025 Jun 9;57(1):2516698. doi: 10.1080/07853890.2025.2516698 (PMC12150644; doi:10.1080/07853890.2025.2516698)
Supplement: Appendix A .docx [file IANN_A_2516698_SM6936.docx]

Appendix A. Standardized differences before and after Inverse Probability Weighting (IPW)

Before IPW

|  | Mean in treated (COVID-19) | Mean in untreated (No COVID-19) | Standardized difference |
| --- | --- | --- | --- |
| Age category | 1.60 | 1.49 | 0.214 |
| Female | 0.50 | 0.48 | 0.040 |
| Race/Ethnicity category | 4.10 | 4.05 | 0.034 |
| Medicaid | 0.27 | 0.34 | -0.152 |
| Baseline comorbid conditions |  |  |  |
| Sleep disorder | 0.04 | 0.02 | 0.109 |
| ADHD | 0.09 | 0.06 | 0.110 |
| Anxiety disorder | 0.14 | 0.08 | 0.201 |
| Depression | 0.07 | 0.04 | 0.169 |
| Chronic pain | 0.22 | 0.13 | 0.241 |
| Injury | 0.13 | 0.08 | 0.170 |
| Suicidal ideation | 0.01 | 0.00 | 0.071 |
| Self-harm | 0.01 | 0.01 | 0.065 |
| Poisoning | 0.00 | 0.00 | 0.038 |
| Obesity | 0.03 | 0.02 | 0.055 |
| Cancer | 0.07 | 0.05 | 0.107 |
| Asthma | 0.08 | 0.04 | 0.151 |
| Chronic  headache/migraine | 0.05 | 0.03 | 0.115 |
| Any allergy | 0.10 | 0.07 | 0.123 |
| Neurologic disorders | 0.12 | 0.08 | 0.115 |

After IPW

|  | Mean in treated (COVID-19 | Mean in untreated (No COVID-19) | Standardized difference |
| --- | --- | --- | --- |
| Age category | 1.52 | 1.52 | -0.002 |
| Female | 0.48 | 0.48 | -0.004 |
| Race/Ethnicity category | 4.07 | 4.06 | 0.001 |
| Medicaid | 0.32 | 0.32 | -0.003 |
| Baseline comorbid conditions |  |  |  |
| Sleep disorder | 0.03 | 0.03 | 0.003 |
| ADHD | 0.07 | 0.07 | 0.006 |
| Anxiety disorder | 0.09 | 0.09 | 0.004 |
| Depression | 0.05 | 0.05 | 0.002 |
| Chronic pain | 0.15 | 0.15 | 0.002 |
| Injury | 0.09 | 0.09 | 0.003 |
| Suicidal ideation | 0.01 | 0.01 | -0.002 |
| Self-harm | 0.01 | 0.01 | 0.005 |
| Poisoning | 0.00 | 0.00 | 0.006 |
| Obesity | 0.02 | 0.02 | 0.002 |
| Cancer | 0.06 | 0.06 | 0.002 |
| Asthma | 0.05 | 0.05 | 0.003 |
| Chronic  headache/migraine | 0.03 | 0.03 | 0.003 |
| Any allergy | 0.08 | 0.08 | 0.004 |
| Neurologic disorders | 0.10 | 0.09 | 0.006 |
